# Attitudes and Learning through Practice Are Key to Delivering Brief Interventions for Heavy Drinking in Primary Health Care: Analyses from the ODHIN Five Country Cluster Randomized Factorial Trial

**DOI:** 10.3390/ijerph14020121

**Published:** 2017-01-26

**Authors:** Peter Anderson, Eileen Kaner, Myrna Keurhorst, Preben Bendtsen, Ben van Steenkiste, Jillian Reynolds, Lidia Segura, Marcin Wojnar, Karolina Kłoda, Kathryn Parkinson, Colin Drummond, Katarzyna Okulicz-Kozaryn, Artur Mierzecki, Miranda Laurant, Dorothy Newbury-Birch, Antoni Gual

**Affiliations:** 1 Institute of Health and Society, Newcastle University, Newcastle NE1 7RU, UK; eileen.kaner@newcastle.ac.uk (E.K.); kathryn.parkinson@newcastle.ac.uk (K.P.); 2 Department of Family Medicine, Maastricht University, Maastricht 6200, The Netherlands; ben.vansteenkiste@maastrichtuniversity.nl; 3 Radboud University Medical Center, Radboud Institute for Health Sciences, Scientific Institute for Quality of Healthcare (IQ healthcare), Nijmegen 6525, The Netherlands; myrna.keurhorst@radboudumc.nl (M.K.); Miranda.Laurant@radboudumc.nl (M.L.); 4 Centre for Nursing Research, Saxion University of Applied Sciences, Deventer 7513, The Netherlands; 5 Department of Medical Specialist, Linköping University, Motala 58183, Sweden; preben.bendtsen@liu.se; 6 Department of Medicine and Health, Linköping University, Motala 58183, Sweden; 7 Psychiatry Determent, Neurosciences Institute, Hospital Clínic, IDIBAPS, Barcelona 08003, Spain; TGUAL@clinic.cat (L.S.); jillianmreynolds@yahoo.com (A.G.); 8 Program on Substance Abuse, Public Health Agency, Government of Catalonia, Barcelona 08003, Spain; lidia.segura@gencat.cat; 9 Department of Psychiatry, Medical University of Warsaw, Warsaw 02-091, Poland; marcin.wojnar@wum.edu.pl; 10 Independent Laboratory of Family Physician Education, Pomeranian Medical University, Szczecin 70-204, Poland; wikarla@gazeta.pl (K.K.); roklr@sci.pam.szczecin.pl (A.M.); 11 National Addiction Centre, Institute of Psychiatry, King’s College London, London WC2B 5RL, UK; colin.drummond@kcl.ac.uk; 12 National Institute for Health Research Biomedical Research Centre for Mental Health, Maudsley NHS Foundation Trust, London SE5 8AF, UK; 13 State Agency for Prevention of Alcohol-Related Problems, Warsaw 59620-2905, Poland; katarzyna.okulicz@parpa.pl; 14 Faculty of Health and Social Studies, HAN University of Applied Sciences, Nijmegen 6525, The Netherlands; 15 Health and Social Care Institute, Teesside University, Middesbrough TS1 3BX, UK; d.newbury-birch@tees.ac.uk

**Keywords:** primary health care, heavy drinking, screening and brief advice, training and support, financial reimbursement, role security, therapeutic commitment, short alcohol and alcohol problems perception questionnaire

## Abstract

In this paper, we test path models that study the interrelations between primary health care provider attitudes towards working with drinkers, their screening and brief advice activity, and their receipt of training and support and financial reimbursement. Study participants were 756 primary health care providers from 120 primary health care units (PHCUs) in different locations throughout Catalonia, England, The Netherlands, Poland, and Sweden. Our interventions were training and support and financial reimbursement to providers. Our design was a randomized factorial trial with baseline measurement period, 12-week implementation period, and 9-month follow-up measurement period. Our outcome measures were: attitudes of individual providers in working with drinkers as measured by the Short Alcohol and Alcohol Problems Perception Questionnaire; and the proportion of consulting adult patients (age 18+ years) who screened positive and were given advice to reduce their alcohol consumption (intervention activity). We found that more positive attitudes were associated with higher intervention activity, and higher intervention activity was then associated with more positive attitudes. Training and support was associated with both positive changes in attitudes and higher intervention activity. Financial reimbursement was associated with more positive attitudes through its impact on higher intervention activity. We conclude that improving primary health care providers’ screening and brief advice activity for heavy drinking requires a combination of training and support and on-the-job experience of actually delivering screening and brief advice activity.

## 1. Introduction

Primary health care providers find screening and giving brief advice for heavy drinking a difficult business [1,2,3]. This can be changed with professional and organizational-based interventions [4,5]. During the 1970s, the Maudsley Alcohol Pilot Project was set up in the United Kingdom to make practical recommendations for an improved local response to dealing with drinking problems [6]. The project, which subsequently informed the United Kingdom’s Royal College of General Practitioners’ report on alcohol [7], was premised on the view that to respond to drinking problems adequately, primary health care providers need to be involved.

The Maudsley Alcohol Pilot Project used the Alcohol and Alcohol Problems Perception Questionnaire (AAPPQ) as a theoretical basis to understand why community agents have difficulty with alcohol problems, and as a basis to monitor improvement [8,9]. In the AAPPQ, for which a shortened version is available [10,11], role security measures role adequacy, for example, “I feel I can appropriately advise my patients about drinking and its effects”; and role legitimacy, for example, “I feel I have the right to ask patients questions about their drinking when necessary”. Role insecurity is expressed at the emotional level as therapeutic commitment, which measures motivation, for example, “pessimism is the most realistic attitude to take toward drinkers”; task specific self-esteem, for example, “all in all I am inclined to feel I am a failure with drinkers”; and work satisfaction, for example, “in general, it is rewarding to work with drinkers”.

The Maudsley Alcohol Pilot Project found that primary health care providers often failed to recognize and respond to drinking problems because they felt anxieties about their role adequacy through not having the information and skills necessary to recognize and respond to drinkers; and because they felt anxieties about their role legitimacy through being uncertain as to whether or indeed how far drinking problems came within their responsibilities. The project proposed that the key to increasing on-the-job experience and effectiveness was to provide education and training to primary health care providers along with supporting brief advice activity, such as referral opportunities, to improve their role security and therapeutic commitment, which, in turn, would lead to more brief advice activity. 

Although there have been a number of international studies examining providers’ attitudes in this field [12,13,14,15,16], as far as we are aware, there have been no published studies with repeated attitude measures that have further examined the original model of the Maudsley Alcohol Pilot Project. The European ODHIN (Optimizing delivery of health care interventions) project reported in this paper allows the opportunity to do so. ODHIN collected data on providers’ role security and therapeutic commitment, and on their screening and brief advice activity, at three separate time points over a 9-month period, including measurements at baseline, during a 12-week implementation period and at a 9-month follow-up. ODHIN also collected data enabling an assessment of the impact of training and support and of financial reimbursement on providers’ attitudes and on their screening and brief advice activity.

**Figure 1:**
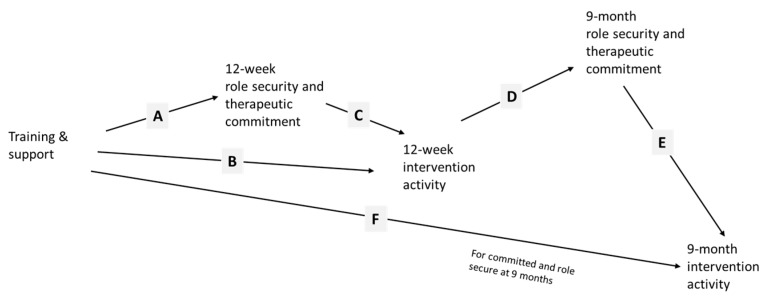
Hypothesized path model for training and support.

**Figure 2:**
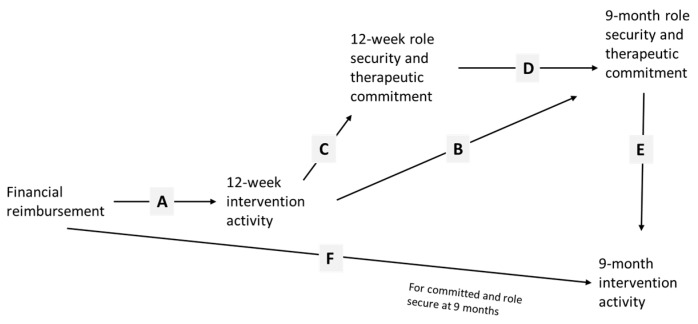
Hypothesized path model for financial reimbursement.

In this current paper, we test path models illustrated in Figure 1 and Figure 2. We aim to demonstrate the importance of on-the-job experience, through improving role security and therapeutic commitment, in leading to more screening and brief advice activity. We also aim to test whether or not financial reimbursement has an enduring effect beyond the time of reimbursement, through its initial impact in improving on-the-job experience.

In Figure 1, we hypothesized that training and support would be associated with improved 12-week role security and therapeutic commitment (line A); that training and support would directly (line B) and indirectly (lines A and C), be associated with increased 12-week screening and advice activity, hereafter termed intervention activity. In turn, we hypothesized that increased 12-week intervention activity would be associated with further improvement in role security and therapeutic commitment (line D), which should subsequently be associated with more 9-month intervention activity (line E). In addition, we hypothesized that training and support would be preferentially associated with increased 9-month intervention activity in those with higher 9-month role security and therapeutic commitment (line F).

Our model should further imply, as in Figure 2, that, whilst financial reimbursement should have no direct relationship with role security and therapeutic commitment, it should be associated with increased role security and therapeutic commitment indirectly through its impact on intervention activity (lines A, B, C, and D). Improved role security and therapeutic commitment should then be associated with future intervention activity (line E). Similar to training and support, financial reimbursement should be preferentially associated with increased 9-month intervention activity in those with higher 9-month role security and therapeutic commitment (line F).

## 2. Methods

Details of the trial protocol [17] and the main results of the ODHIN study [5] have been published. In a cluster randomized 2 × 2 × 2 factorial trial, data from primary health care units (PHCUs) in Catalonia, England, The Netherlands, Poland, and Sweden were combined to examine the effect of training and support, financial reimbursement, and referral opportunities to an internet-based advice program (electronic brief intervention, eBI (electronic brief intervention)) on the proportion of consulting adult patients given an intervention (screening and advice to screen positives) for heavy drinking, operationalized by AUDIT-C [18].

### 2.1. Participants

PHCUs with approximately 5000–20,000 registered patients were the unit of randomization and implementation. Individuals from PHCUs who agreed to participate in the study were volunteers drawn from administrative or academic registries of PHCUs at national or regional levels. Eligible providers in each PHCU included any fully trained full- or part-time medical practitioner, nurse, or PHCU assistant with a permanent appointment working in the PHCU and involved in medical and/or preventive care. Providers are the unit of analysis in this paper.

### 2.2. Implementation Strategies

PHCUs were randomized to one of the following eight groups: (1) control; (2) training and support (TS); (3) financial reimbursement (FR); (4) electronic brief intervention (eBI); (5) training and support and financial reimbursement; (6) training and support and eBI; (7) financial reimbursement and eBI; and (8) training and support, financial reimbursement, and eBI. In this paper, we only consider training and support and financial reimbursement (rather than electronic brief intervention), since these were the two implementation strategies, that led to an increase in screening and brief advice activity [5].

PHCUs were asked to screen all adult patients (aged 18 years) for heavy drinking who consulted the PHCU for whatever reason, using a paper version of AUDIT-C, except in Catalonia, where a computerized version was used. Screen positives were defined in Catalonia and England as men and women who scored ≥5 on AUDIT-C, and in Poland, The Netherlands, and Sweden as men who scored ≥5 and women who scored ≥4 on AUDIT-C. PHCUs were asked to deliver brief alcohol advice for 5–15 minutes to screen positive patients. 

### 2.3. Outcomes

Role security and therapeutic commitment of the participating providers were measured using the short version of the Alcohol and Alcohol Problems Perception Questionnaire (SAAPPQ) [10,11]. Measurements took place during the 4-week baseline period, towards the end of the 12-week implementation period, and during the 4-week follow-up period at 9 months. The questionnaire comprised 10 statements, which addressed five subscales: (i) role adequacy; (ii) role legitimacy; (iii) motivation; (iv) task specific self-esteem; and (v) work satisfaction. Responses to the statements were scored from 1 (strongly disagree) to 7 (strongly agree). Scores on the subscales “role adequacy” and “role legitimacy” were added to form an index of “role security” [10], with a total score ranging from 4 to 28. The subscales relating to “self-esteem”, “motivation”, and “work satisfaction” were added to form an index of “therapeutic commitment” [10], with a total score ranging from 6 to 42. Individual missing values for any of the items in a domain were assigned the mean value of the remaining items of the domain before summation. For certain analyses, providers were split into those with baseline scores below and above the mean for role security (mean value 20.98; 45.3% below the mean), and therapeutic commitment (mean value 27.21; 53.6% below the mean); for other analyses, providers were also split into those with 9-month scores below and above the mean for role security (mean value 21.56; 45.8% below the mean), and therapeutic commitment (mean value 27.44; 52.7% below the mean). Providers with scores below the mean are called “role insecure” and “uncommitted”; above the mean, providers are called “role secure” and “committed”.

Screening and brief advice activity were measured at three time points: during the 4-week baseline period, during the 12-week implementation period (when the intervention strategies were actively applied), and during the 4-week follow-up period that occurred at 9 months—6 months after the end of the 12-week implementation period. For each of the measurement periods, the outcome is the proportion of consulting adult patients given an intervention (screened and advice to screen-positives), hereafter termed proportion intervened. Proportion intervened is defined as the number of AUDIT-C positive patients that received one or more of the following items: oral advice; an advice leaflet; referral to the eBI program; or, referral for advice to another provider in or outside the PHCU, divided by the total number of adult consultations of the participating provider. For certain analyses, providers were split into those who, at baseline, did not intervene with any patients (52.3% of sample) and those who, at baseline, intervened with one or more patients. Providers who intervened with no patients at baseline are called “zero intervenors”; providers who intervened with at least one patient at baseline are called greater-than-zero intervenors (“GT zero intervenors”).

### 2.4. Randomization and Blinding

Randomization took place after formal agreement of the PHCU to take part in the trial. The PHCUs were randomly allocated to 1 of the 8 groups by the ODHIN coordinating center, using computerized randomization, stratified by country, ensuring 15 PHCUs per group (3 per group per country).

### 2.5. Sample Size

It was estimated that 56 PHCUs (7 per each of 8 allocation groups) with a minimum of 1000 adult patients consulting per month would be needed for an 80% chance of detecting an increase in the proportion of consulting adult patients, given an intervention from 4% to at least 6% (alpha = 0.05). In calculating our sample size, we used an estimate of intraclass correlation coefficient (ICC) of 0.029 across primary care interventions [19], based on one PHCU study of implementation of alcohol screening and advice [20]. Sample size estimation was conducted using STATA 12 (StataCorp, College Station, TX, USA). As country was used as stratification criteria, each country included a minimum of 24 PHCUs.

### 2.6. Statistical Methods

Two sets of dependent variables were analyzed. The first set were scores for role security and therapeutic commitment per provider; in these analyses, the independent variables were exposure to two of the implementation strategies (training and support and financial reimbursement), and the proportion of patients intervened. The second set of dependent variables were proportion of patients intervened with at different time periods; in these analyses, the independent variables were exposure to two of the implementation strategies (training and support and financial reimbursement), and scores for role security and therapeutic commitment per provider. For all follow-up data, values at previous time points were incorporated as covariates in the models. 

Distributional assumptions of the outcome variables were assessed and natural log transformations were undertaken for the proportion of patients intervened. As this approach creates some issues with outcomes with a zero value, 0.001 was added to each proportion prior to logging.

The study was conceived and analysed as a factorial design, in which (−1, 1) coding was used for the factors, (in this case, training and support and financial reimbursement) resulting in regression coefficients having half the effects. In presenting the results, the estimates for training and support and for financial reimbursement have been doubled. The factorial design is based on the premise that the effect of, for example, training and support instead of no training and support can not only be estimated from TS vs. control, but also from TS + FR vs. FR, TS + eBI vs. eBI, and TS + FR + eBI vs. FR + eBI, giving a pooled estimate with more precision (for definition of abbreviations, see Section 2.2 on implementation strategies). The two factors for the interventions were coded as follows:

TS = −1 for control, FR, eBI, FR + eBI; and + 1 for TS, TS + FR, TS + eBI, TS + FR + eBI; and
FR = −1 for control, TS, eBI, TS + eBI; and + 1 for FR, FR +TS, FR + eBI, FR + TS + eBI

Unstandardized estimates are presented throughout, as there are problems in standardizing the factors, which, in a factorial design are analyzed as continuous variables with a value of either −1.0 or 1.0. A generalized linear model was used, employing a multilevel approach using country and PHCU with random intercepts and slopes. Analysis was conducted using IBM SPSS V22 (IBM, North Castle, NY, USA), procedure MIXED.

## 3. Results

Of the 746 providers at baseline, 408 providers (55%) were doctors, 562 (75%) were women, and the mean age was 46.8 years (SD = 9.3).

### 3.1. Role Security

**Figure 3:**
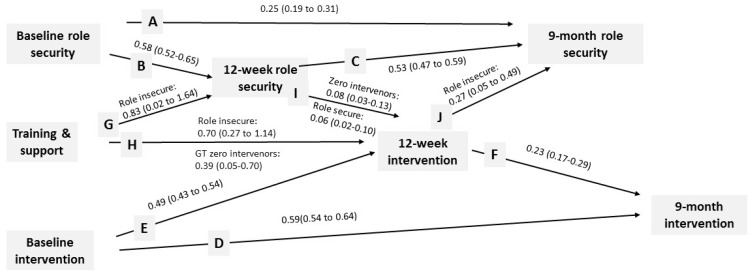
Unstandardized estimates (95% confidence intervals) for interrelationships between role security and the proportion of patients intervened (estimates for impact of training and support have been doubled—see statistical methods).

Figure 3 shows that previous role security was associated with future role security (lines A, B, and C), and that the previous proportion of patients intervened was associated with the future proportion intervened (lines D, E, and F).

Training and support was associated with increased 12-week role security (line G) for those who were role insecure at baseline, for whom role security was one point higher (20.0) with training and support, compared to without (19.0).

Training and support was directly associated with a higher proportion of patients intervened with at 12 weeks (line H) for those who were role insecure at baseline, for whom the proportion was 26/1000 with training and support, compared to 15/1000 without; and for those who had intervened with at least one patient at baseline, for whom the proportion at 12 weeks was 28/1000 with training and support, compared to 19/1000 without. Training and support was indirectly associated with a higher proportion of patients intervened at 12 weeks (lines G and I)—line I for those who were role secure at baseline and for those who had intervened with at least one patient at baseline, for whom, in both cases, there was an increase in the proportion of patients intervened at 12 weeks of 1/1000 for every one-point increase in role security.

The proportion of patients intervened at 12 weeks was associated with a future increase in role security (line J) for those who were role insecure at baseline, for whom role security increased by one point for every 1/100 increase in the proportion intervened at 12 weeks. Role security at 9-month follow-up was not associated with an increase in the proportion of patients intervened at 9 months.

There was no association between training and support and the proportion of patients intervened at 9 months for those with high 9-month role security (hypothesized line F in Figure 1).

### 3.2. Therapeutic Commitment

**Figure 4:**
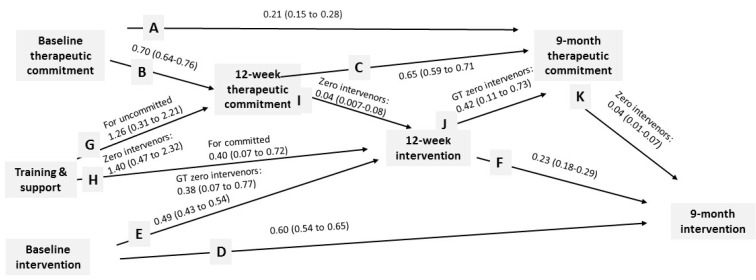
Unstandardized estimates (95% confidence intervals) for interrelationships between therapeutic commitment and proportion of patients intervened (estimates for impact of training and support have been doubled—see statistical methods).

Figure 4 shows that previous therapeutic commitment was associated with future therapeutic commitment (lines A, B, and C), and that the previous proportion of patients intervened was associated with the future proportion intervened (lines D, E, and F). 

Training and support was associated with increased 12-week therapeutic commitment (line G) for those who were uncommitted at baseline, for whom therapeutic commitment was 1.5 points higher (26.0) with training and support than without (24.5); it was also the case for those who had intervened with at least one patient at baseline, for whom, also, therapeutic commitment was 1.5 points higher (28.0) with training and support than without (26.5).

Training and support was directly associated with a higher proportion of patients intervened at 12 weeks (line H) for those who were committed at baseline, for whom the proportion was 18/1000 with training and support, compared to 13/1000 without; and for those who had intervened with at least one patient at baseline, for whom the proportion at 12 weeks was 28/1000 with training and support, compared to 19/1000 without. Training and support was indirectly associated with a higher proportion of patients intervened at 12 weeks (lines G and I)—line I for those who had intervened with at least one patient at baseline, for whom there was an increase in the proportion of patients intervened at 12 weeks of 2/1000 for every one-point increase in therapeutic commitment.

### 3.3. Financial Reimbursement

**Figure 5:**
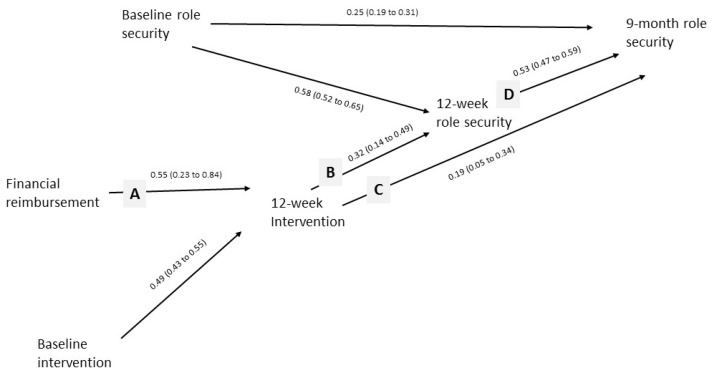
Unstandardized estimates (95% confidence intervals) for interrelationships between role security and financial reimbursement (estimates for impact of financial reimbursement have been doubled—see statistical methods).

Financial reimbursement was associated indirectly with increased 12-week and 9-month role security, Figure 5 (lines A, B, C, and D). Nine-month role security was not associated with the proportion of patients intervened at 9 months. There was no association between financial reimbursement and the proportion of patients intervened at 9 months for those with high 9-month role security (hypothesized line F in Figure 2).

**Figure 6:**
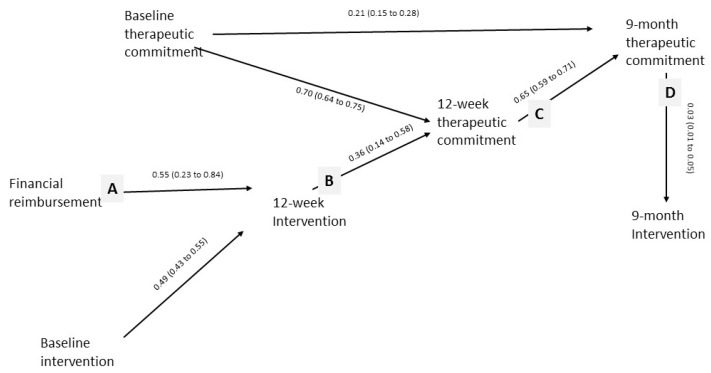
Unstandardized estimates (95% confidence intervals) for interrelationships between therapeutic commitment and financial reimbursement (estimates for impact of financial reimbursement have been doubled—see statistical methods).

Financial reimbursement was associated indirectly with increased 12-week therapeutic commitment, Figure 6 (lines A and B). Twelve-week therapeutic commitment was associated with increased 9-month therapeutic commitment (line C), which, in turn, was associated with a higher proportion of patients intervened at 9 months (line D). As predicted by the hypothesized path model of Figure 2 (line F), financial reimbursement was directly associated with a higher proportion of patients intervened at 9 months for committed (estimate = 0.26, 95% CI = 0.008–0.52) but not for uncommitted providers at 9 months.

## 4. Discussion

Numerous systematic reviews and meta-analyses have concluded that there is a positive impact of primary health care-based screening and brief advice programs in reducing heavy drinking [21,22,23]. Although the extent to which this evidence-base can be interpreted as efficacy (ideal world) or effectiveness (real world) trials is still debated in some academic circles [24,25], nevertheless, screening and brief alcohol intervention programs are recommended as good practice for preventive care in primary care systems worldwide [18,26,27,28]. Modeling studies have suggested that were these programs widely implemented, considerable population health gain could be achieved [28]. The public health problem is one of failure to achieve widespread take-up of screening and brief advice programs [29]. In the ODHIN trial, we found that only 5.9% of eligible patients consulting their primary health care provider during a 4-week baseline measurement period were screened for their alcohol consumption [5]. ODHIN demonstrated that up to 4 h training and support to primary health care providers and financial reimbursement delivered during a 12-week implementation period resulted in a higher proportion of heavy drinkers given a brief intervention (screened and advice given to screen-positives) to reduce their drinking [5]. The ratio of the logged proportion given an intervention during the 12-week implementation period was 1.61 (95% CI = 1.24–2.10) in primary care units that received training and support versus those that did not receive training and support; for financial reimbursement, the ratio was 2.00 (95% CI = 1.49–2.47). This present paper has examined the extent to which on-the-job experience, through improving role security and therapeutic commitment, leads to more screening and brief advice activity. The paper also examined whether or not financial reimbursement had an enduring effect beyond the time of reimbursement, through its short-term impact in improving on-the-job experience.

We report associations over time between: primary health care providers’ attitudes to managing heavy drinking patients; their exposure to factors aimed at increasing their screening and brief advice activity for heavy drinking; and changes in the proportion of providers’ adult patients who were screened and offered advice to reduce their heavy drinking. Inevitably, if associations are found, they may operate in two directions. For example, if increases in the proportion of patients screened and advised are found (independent variable) to be associated with increases in positive attitudes (dependent variable), we are likely to find an association between increases in positive attitudes (independent variable) and increases in the proportion of patients screened and advised (dependent variable), which we do. However, because we repeated measures over time, we are able to tease out some of the likely directions of the relationships.

Our findings are in line with the model put forward by the Maudsley Alcohol Pilot Project [6]. Through direct and indirect (via changes in attitudes) paths, training and support was associated with improved screening and brief advice activity during the 12-week implementation period. Twelve-week screening and advice activity was associated with future role security and therapeutic commitment. Nine-month therapeutic commitment, but not role security, was associated with increased nine-month screening and advice activity. The model is further corroborated by the demonstration that, whilst financial reimbursement was not directly associated with improvements in role security and therapeutic commitment, through its association with increased 12-week screening and advice activity, it was indirectly associated with improved future role security and therapeutic commitment. 

A criticism of pay for performance is that changed behavior is unlikely to persist once the incentive is removed. However, our findings suggest that it could be possible for financial reimbursement to have an impact beyond removal of the incentive. Financial reimbursement was associated with increased screening and brief advice activity, which in turn was associated with improved role security and therapeutic commitment, with increased therapeutic commitment, in turn, associated with increased screening and brief advice activity beyond the duration of the reimbursement. Thus, financial incentives can open the door to positive changes in practitioners’ screening and advice activity, which then are likely to be reinforced by day-to-day repetition (familiarity) and embedding of the action in routine practice behavior. Future withdrawal of pay for performance schemes, thus, may not extinguish the desired practitioner behavior. Financial reimbursement may be more useful, though, if it encourages embedding approaches (such as templates or registers), as occurs in the quality and outcomes framework, part of the General Medical Services contract for general practices in England [30]. 

## 5. Conclusions

Our findings suggest that to improve primary health care providers’ screening and brief advice activity for heavy drinking, two actions are needed: first, the provision of good quality training and support to all providers, irrespective of initial levels of role security and therapeutic commitment, and irrespective of initial screening and brief advice activity; and, second, on-the-job experience, by actually delivering screening and brief advice activity. Screening and brief advice activity may be further enhanced when embedded within wider policy and community actions [26,31].
